# *ETV6*::*RUNX1* Acute Lymphoblastic Leukemia: how much therapy is needed for cure?

**DOI:** 10.1038/s41375-024-02287-7

**Published:** 2024-06-06

**Authors:** Anna Østergaard, Marta Fiocco, Hester de Groot-Kruseman, Anthony V. Moorman, Ajay Vora, Martin Zimmermann, Martin Schrappe, Andrea Biondi, Gabriele Escherich, Jan Stary, Chihaya Imai, Toshihiko Imamura, Mats Heyman, Kjeld Schmiegelow, Rob Pieters

**Affiliations:** 1 Princess Máxima Center for Pediatric Oncology, Utrecht, The Netherlands; 2 Mathematical Institute, Leiden University, Leiden, The Netherlands; 3 Department of Biomedical Science, Section Medical Statistics, Leiden University Medical Center, Leiden, The Netherlands; 4 Dutch Childhood Oncology Group (DCOG), Utrecht, The Netherlands; 5 Leukaemia Research Cytogenetics Group, Translational and Clinical Research Institute, Newcastle University, Newcastle upon Tyne, UK; 6 United Kingdom Acute Lymphoblastic Leukaemia (UKALL) study group, Liverpool, UK; 7 Department of Haematology, Great Ormond Street Hospital, London, UK; 8 Department of Paediatric Haematology and Oncology, Hannover Medical School, 30625 Hannover, Germany; 9 Berlin-Frankfurt-Münster Study Group (BFM), Frankfurt, Germany; 10 Department of Paediatrics, University Medical Centre Schleswig-Holstein, Kiel, Germany; 11 Department of Pediatrics, University of Milano-Bicocca, Monza, Italy; 12 Associazione Italiana di Ematologia e Oncologia Pediatrica (AIEOP), Bologna, Italy; 13 Department of Pediatric Hematology and Oncology, University Medical Center Hamburg-Eppendorf, Hamburg, Germany; 14 Childhood Acute Lymphoblastic Leukemia study group (CoALL), Hamburg, Germany; 15 Department of Pediatric Hematology and Oncology, Second Faculty of Medicine, Charles University, Prague, Czech Republic; 16 Department of Pediatrics, Niigata University Graduate School of Medical and Dental Sciences, Niigata, Japan; 17 Children’s Cancer and Leukemia Study Group (CCLSG), Nagoya, Japan; 18 Department of Pediatrics, Kyoto Prefectural University of Medicine, Graduate School of Medical Science, Kyoto, Japan; 19 Japan Childhood Leukemia Study Group (JACLS), Nagoya, Japan; 20 Childhood Cancer Research Unit, Department of Women’s and Children’s Health, Karolinska Institutet, Stockholm, Sweden; 21 Department of Paediatric Oncology, Karolinska University Hospital, Stockholm, Sweden; 22 Nordic Society of Paediatric Haematology and Oncology (NOPHO), Nordic Countries, Uppsala, Sweden; 23 Department of Pediatrics and Adolescent Medicine, University Hospital Rigshospitalet, Institute of Clinical Medicine, Faculty of Medicine, University of Copenhagen, Copenhagen, Denmark; 24 Nordic Society of Paediatric Haematology and Oncology (NOPHO), Nordic and Baltic Countries, Uppsala, Sweden

**Keywords:** Epidemiology, Chemotherapy

## Abstract

Recent trials show 5-year survival rates >95% for *ETV6*::*RUNX1* Acute Lymphoblastic Leukemia (ALL). Since treatment has many side effects, an overview of cumulative drug doses and intensities between eight international trials is presented to characterize therapy needed for cure. A meta-analysis was performed as a comprehensive summary of survival outcomes at 5 and 10 years. For drug dose comparison in non-high risk trial arms, risk group distribution was applied to split the trials into two groups: trial group A with ~70% (range: 63.5–75%) of patients in low risk (LR) (CCLSG ALL2004, CoALL 07-03, NOPHO ALL2008, UKALL2003) and trial group B with ~45% (range: 38.7–52.7%) in LR (AIEOP-BFM ALL 2000, ALL-IC BFM ALL 2002, DCOG ALL10, JACLS ALL-02). Meta-analysis did not show evidence of heterogeneity between studies in trial group A LR and medium risk (MR) despite differences in treatment intensity. Statistical heterogeneity was present in trial group B LR and MR. Trials using higher cumulative dose and intensity of asparaginase and pulses of glucocorticoids and vincristine showed better 5-year event-free survival but similar overall survival. Based on similar outcomes between trials despite differences in therapy intensity, future trials should investigate, to what extent de-escalation is feasible for *ETV6*::*RUNX1* ALL.

## Introduction

B-cell acute lymphoblastic leukemia (B-ALL) is the most common childhood malignancy and is classified according to genetic aberrations [1, 2]. Around 25% of cases harbor a translocation t(12;21) leading to an *ETV6*::*RUNX1* fusion gene [3]. Recent treatment protocols show excellent results in this cytogenetic subgroup with 5-year survival rates above 95% [4–6]. However, treatment has significant short [6, 7] - and long-term side effects [8–11]. Since current multiagent strategies of various study groups consist of nearly identical drugs [4–6, 12–16], a comparison of drug dosage and treatment intensity of trials can lead to more insight into how much therapy is needed, and which components of treatment are necessary for cure. This may indicate which toxic therapy elements can be reduced in future trials. Cumulative drug dose and treatment intensity of eight clinical trials of study groups participating in the I-BFM ALL network were compared. In addition, a meta-analysis of survival outcomes (cumulative incidence of relapse, event-free and overall survival, death in complete remission) was performed to provide a comprehensive summary.

## Methods

### Included clinical trials

**Fig. 1:**
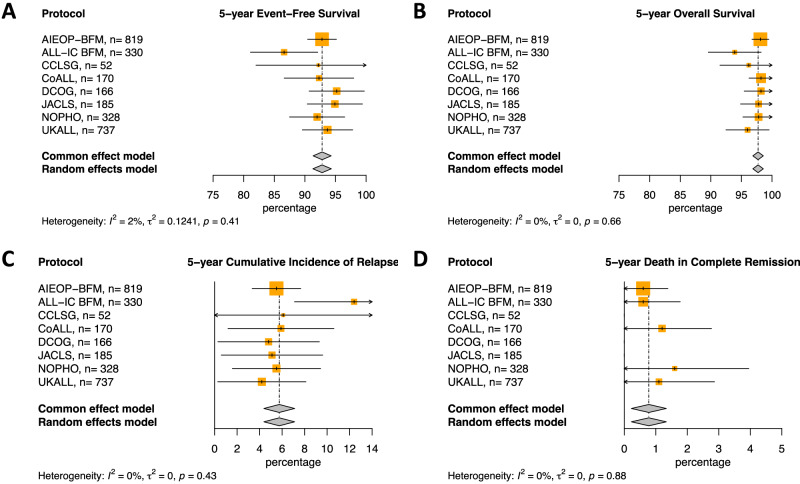
Meta-analysis of outcome of LR and MR arms of trials included in *ETV6*::*RUNX1* therapy intensity study. **A** 5-year event-free survival (%), **B** 5-year overall survival (%), **C** 5-year cumulative incidence of relapse (%), **D** 5-year cumulative incidence of death in remission (%). EFS event-free survival, OS overall survival, CIR clinical incidence of relapse, DCR death in complete remission, CCLSG Children’s Cancer and Leukemia Study Group, CoALL Childhood Acute Lymphoblastic Leukemia, NOPHO Nordic Society of Pediatric Hematology and Oncology, UKALL United Kingdom Acute Lymphoblastic Leukemia; AIEOP-BFM, Associazione Italiana di Ematologia e Oncologia Pediatrica and Berlin Frankfurt Münster; ALL IC-BFM Acute Lymphoblastic Leukemia Intercontinental-Berlin Frankfurt Münster, DCOG Dutch Childhood Oncology Group, JACLS Japan Childhood Leukemia Study Group.

Eight national or collaborative group clinical trials contributed to this study: Children’s Cancer and Leukemia Study Group (CCLSG, Japan) ALL2004, Childhood Acute Lymphoblastic Leukemia (CoALL, Germany) 07-03, Nordic Society of Pediatric Hematology and Oncology (NOPHO, Nordic and Baltic countries) ALL2008, United Kingdom Acute Lymphoblastic Leukemia (UKALL, United Kingdom) 2003, Associazione Italiana di Ematologia e Oncologia Pediatrica and Berlin Frankfurt Münster (AIEOP-BFM, Germany, Italy, Austria, Switzerland) ALL 2000, Acute Lymphoblastic Leukemia Intercontinental-Berlin Frankfurt Münster (ALL IC-BFM, Argentina, Chile, Croatia, Cuba, Czech Republic, Hong Kong, Hungary, Israel, Poland, Serbia, Slovakia, Slovenia, Ukraine, Uruguay, Moscow) ALL 2002, Dutch Childhood Oncology Group (DCOG, The Netherlands) ALL10 and Japan Childhood Leukemia Study Group (JACLS, Japan) ALL-02 (Fig. 1A). Patients 1–18 years of age were enrolled in the trials between 2000 and 2014. All participating countries approved the treatment protocols according to national law and guidelines. Informed consent was obtained according to the Declaration of Helsinki. Participating study groups, clinical trials, and the number of patients in each trial are specified in Table 1. Study groups were asked to send trial protocols and fill out a data collection table with aggregated survival data and cumulative drug doses on patients with cytogenetically proven *ETV6*::*RUNX1* ALL. Aggregated survival data at 5 and 10 years could be obtained from the participating study groups, however survival curves could not be obtained from all. All protocols consisted of induction, consolidation, intensification and maintenance chemotherapy courses, and central nervous system (CNS) directed therapy [4–6, 12–16]. Risk stratification criteria and use of minimal residual disease (MRD) differed between trials. Therefore, study risk groups were defined, and the risk stratification of the individual trials was categorized within these study risk groups (Table 1). Four trials used MRD although not all used the same method of quantification nor the same cut-off for risk stratification leading to different risk group distributions. For each trial, the arm(s) with up to 10% of the highest risk patients were categorized as high risk (mean 2.7% of all *ETV6*::*RUNX1* patients, range 0–9.7%). This small group of high-risk patients was not analyzed further. Of the remaining arms including 97.3% of all *ETV6*::*RUNX1* patients, the most intense arm in each study group was categorized as medium risk (MR) and the least intense as low risk (LR) (Table 1).

**Table 1:** *ETV6*::*RUNX1* therapy intensity study risk group definition.

Study group	Clinical trial	Total number of patients	Risk group in *ETV6*::*RUNX1* study			Risk group distribution			5-year outcome of LR and MR arms				MRD technique	LR stratification criteria	MR stratification criteria	HR stratification criteria
			LR	MR	HR	LR	MR	HR	EFS	OS	CIR	DCR				
						*n* (%)	*n* (%)	*n* (%)	% (SE)	% (SE)	% (SE)	% (SE)				
*Group A*																
CCLSG	ALL2004	56	SR	HR	HHR + SR salvage	42 (75)	10 (17.9)	4 (7.1)	92.2 (5.2)	96.2 (2.4)	6.1 (4.8)	0	PCR	1-9 years, WBC < 50	<1 or >9 years or WBC 50-100	WBC > 100 or MRD > 10–3 at day 77
CoALL	07-03	170	SR	HR		108 (63.5)	62 (36.5)	0 (0)	92.3 (2.9)	98.2 (1.0)	5.9 (2.4)	1.2 (0.8)	–	<10 years, WBC < 25 and good in vitro drug response	≥10 years, WBC ≥ 25 or poor in vitro drug response	
NOPHO	ALL2008	333	SR	IR	HR	234 (70.3)	94 (28.2)	5 (1.5)	92.0 (2.3)	97.8 (1.3)	5.5 (2.0)	1.6 (1.2)	Flowcytometry	MRD < 0.1% at EOI	MRD > 0.1% and <5% at EOI	MRD ≥ 5% at EOI or ≥ 0.01% at day 79
UKALL	2003	736	A	B + C		519 (70.4)	218 (29.6)	0 (0)	93.7 (2.1)	96.0 (1.8)	4.2 (2.0)	1.1 (0.9)	PCR	MRD < 0.01% at EOI	MRD > 0.01% at EOI	
*Group B*																
AIEOP-BFM	ALL 2000	843	SR	MR	HR	444 (52.7)	375 (44.5)	24 (2.8)	92.8 (1.2)	98.1 (0.7)	5.5 (1.1)	0.6 (0.4)	PCR	MRD negative at EOI	MRD positive at EOI, <5 × 10-4 at day 78	MRD > 5 × 10–4 at day 78, Poor prednisolone response
ALL-IC BFM	ALL 2002	352	SR	MR	HR	151 (42.9)	179 (50.8)	22 (6.3)	86.6 (2.8)	93.9 (2.2)	12.4 (2.7)	0.6 (0.6)	–	1–6 years, WBC < 20×10-9/L, <5% blasts at EOI	≥6 years and <5% blasts at EOI	≥5% blasts at EOI, Poor prednisolone response
DCOG	ALL10	168	SR	MR	HR	65 (38.7)	101 (60.1)	2 (1.2)	95.2 (2.3)	98.2 (1.4)	4.8 (2.3)	0	PCR	MRD negative at EOI	MRD positive at EOI, <10-3 at day 79	MRD > 10-3 at day 79, Poor prednisolone response
JACLS	ALL-02	205	SR	HR	ER + F	93 (45.4)	92 (44.9)	20 (9.7)	94.9 (2.3)	97.8 (1.5)	5.1 (2.3)	0	–	<10 years, WBC < 10	≥ 10 years, WBC ≥ 10	Poor prednisolone response

*MRD* minimal residual disease, *EOI* end of induction, *CCLSG* Children’s Cancer and Leukemia Study Group, *CoALL* Childhood Acute Lymphoblastic Leukemia, *NOPHO* Nordic Society of Pediatric Hematology and Oncology, *UKALL* United Kingdom Acute Lymphoblastic Leukemia, *AIEOP-BFM* Associazione Italiana di Ematologia e Oncologia Pediatrica and Berlin Frankfurt Münster, *ALL IC-BFM* Acute Lymphoblastic Leukemia Intercontinental-Berlin Frankfurt Münster, *DCOG* Dutch Childhood Oncology Group, *JACLS* Japan Childhood Leukemia Study Group, *SR* standard risk, *LR* low risk, *MR* medium risk, *IR* intermediate risk, *HR* high risk, *HHR* very high risk, *ER* extremely high risk, *F* failure, *EFS* event-free survival, *OS* overall survival, *CIR* clinical incidence of relapse, *DCR* death in complete remission, *PCR* polymerase chain reaction.

### Calculation of cumulative drug dose

Cumulative doses were calculated by multiplying the prescribed dose in mg/m^2^ by the number of administrations specified in the trial protocol. To group glucocorticoids together, doses of dexamethasone were multiplied by 6.7 to account for its greater anti-leukemic effect compared to prednisone [17–19]. The cumulative doses of anthracyclines (doxorubicin, daunorubicin, pirarubicin) and thiopurines (6-mercaptopurine and 6-thioguanine) were grouped together in a one-to-one ratio [4, 11].

The effect of asparaginase depends on the duration of asparagine depletion in leukemic cells [20, 21]. Therefore, Native E-coli Asparaginase was classified as three days of asparagine depletion per dose, and pegylated Asparaginase as 14 days [22]. Cumulative methotrexate (MTX) doses were separated in intravenous high dose ( > 90 mg/m^2^) (HD-MTX) and maintenance oral low dose (LD-MTX) administration [23, 24].

Intrathecal injections have specific doses according to the age of the patient [6, 14] and is therefore assessed by number of intrathecal administrations (both triple [MTX, cytarabine, glucocorticosteroid] and MTX only).

Cumulative doses are reported per study risk group for each trial. When randomizations were present, mean cumulative doses were calculated based on number of patients in each arm. When comparing trials, cumulative doses are presented relative to the doses used in the largest trial in the trial group. Using 0.8–1.2 times the dose in the reference trial was defined as similar, <0.8 times the reference dose was defined as lower and >1.2 times as higher. Absolute cumulative doses are presented in Table 2.

**Table 2:** Cumulative doses of drugs used in clinical trials included in *ETV6*::*RUNX1* therapy intensity study.

Clinical trial	Treatment arm	Study risk group	*N* (% of total)	Glucocorticoids^a^	Vincristine^a^	Asparaginase^b^	Thiopurines^a^	HD-MTX^a^	LD-MTX^a^	Anthracyclines^a^	Cyclophosphamide^a^	Intrathecal administrations^c^	Cytarabine^a^	Treatment length^d^
*Group A*														
CCLSG ALL2004	SR	LR	42 (75)	12230	52	21	33750	12000	4500	115	3200	16	3200	110
CCLSG ALL2004	HR	MR	10 (17.9)	17706	80	35	45860	27000	5175	505	6800	22	7200	165
CoALL 07-03	SR	LR	108 (63.5)	2618	9	8	26450	3000	1600	204	900	10	12660	104
CoALL 07-03	HR	MR	62 (36.5)	3556	12	10	22800	4000	1600	264	3600	12	24600	104
NOPHO ALL2008	SR	LR	234 (70.3)	3810	32	24	60890	40000	2240	80	0	13	600	130
NOPHO ALL2008	IR	MR	94 (28.2)	4815	40	24	57005	40000	2060	200	2000	24	1200	130
UKALL2003	A	LR	519 (70.4)	8699	60	7	65736	0	2388	122	1632	21	979	141
UKALL2003	B + C	MR	218 (29.6)	8613	67	11	64455	667	2258	225	3673	23	2204	139
*Group B*														
AIEOP-BFM ALL 2000	SR	LR	444 (52.7)	2926	10.5	5.5	30020	20000	1492	210	2750	11	1800	104
AIEOP-BFM ALL 2000	MR	MR	375 (44.5)	3395	12	6.3	30440	20000	1444	240	3000	12	2100	104
ALL-IC BFM 2002	SR	LR	151 (42.9)	3322	12	6.3	30415	8000	1490	180	3000	15	2100	104
ALL-IC BFM 2002	IR	MR	179 (50.8)	3790	13.5	6.6	29715	8000	1450	270	3250	15	2400	104
DCOG ALL10	SR	LR	65 (38.7)	2618	9	5.5	31080	20000	1600	120	2000	9	1200	104
DCOG ALL10	MR	MR	101 (60.1)	7308	62	33.5	30030	20000	1860	300	2000	14	1200	104
JACLS ALL-02	SR	LR	93 (45.4)	8034	42	5	31150	6000	2200	100	1500	11	900	102
JACLS ALL-02	HR	MR	92 (44.9)	6354	48	25	11200	6000	3600	300	7600	14	3600	98

Trials are grouped based on risk group distribution: group A with ~70% (range: 63.5–75%) and group B with ~45% (range: 38.7–52.7%) of patients in the low risk arm.

*SR* standard risk, *LR* low risk, *IR* intermediate risk, *MR* medium risk, *HR* high risk, *HD-MTX* high-dose methotrexate, *LD-MTX* low-dose methotrexate. ^a^mg/m^2^, ^b^weeks of asparagine depletion, ^c^number of intrathecal administrations, ^d^number of weeks of treatment.

### Calculation of treatment intensity

Treatment intensity was calculated by a modification of the method described by Hryniuk et al. [25–27]. Three treatment periods were assessed: (i) day 0–90, where toxic death is most common [28], (ii) day 0 till start of maintenance therapy, and (iii) maintenance therapy. For each period, the cumulative dose of a single drug was divided by the number of weeks resulting in a weekly dose of each drug. This was divided by the weekly dose administered in the reference trial resulting in a single drug relative dose intensity. To calculate the combined relative dose intensity of a treatment period, single drug intensities were added up and divided by the total number of drugs used (Fig. S1). If a certain drug was not administered during the treatment period in question, the relative dose intensity was set to zero. When randomizations were present, mean dose intensities were calculated based on number of patients in each arm.

### Statistical analysis

Estimates of 5- and 10-year event-free survival (EFS), overall survival (OS), cumulative incidence of relapse (CIR) and death in complete remission (DCR) were the endpoints of interest for each study. An event was defined as either no complete remission (by resistant disease or death during induction), relapse, second malignant neoplasm or death due to any cause. When data on survival outcome at 5 or 10 years of at least three protocols was available, both fixed and random model were employed to pool study-specific survival outcomes in order to estimate an overall survival outcome and its associated confidence intervals. The overall effect estimated with a fixed and random effects model are reported. The sizes of the square boxes on the forest plot are proportional to the total number of *ETV6*::*RUNX1* patients in the specific study. An overall test on heterogeneity between studies was performed for each separate meta-analysis (value I-squared in figures). To estimate the between-study variance which is represented as ‘tau’ in the forest plots, DerSimonian-Laird’s method has been employed [29]. All aggregated survival outcomes employed in the meta-analysis model were provided by the individual study groups. The meta-analysis has been performed in R version 4.3.0 environment with the library meta version 7.0-0 [30, 31].

## Results

In total, 2864 patients with an *ETV6*::*RUNX1* translocation were treated in the 8 trials, of which 2787 (97.3%) patients were stratified as low risk (LR) or medium risk (MR) (Table 1). Results from the meta-analysis applied to the LR and MR 5- and 10-year survival outcomes show no evidence of heterogeneity between trials (Table 1, Figs. 1A, 1B, 1C, 1D, S2, S3). This means that results and findings across the individual trials included in the analysis are consistent with each other. EFS at 5- and 10-years was > 90% for all trials except for ALL-IC BFM ALL 2002 and JACLS ALL-02 while 5- and 10-year OS was > 95% for all trials except ALL-IC BFM and CCLSG (Table 1, Figs. 1A, 1B, 1C, 1D, S2, S3).

Due to differences in stratification criteria (Table 1) the eight clinical trials differed in their risk group distribution. Hence the trials were split into two trial groups, those with ~70% of patients in LR (range: 63.5–75%) and ~30% in MR (range: 17.9–36.5%) (trial group A) and those with ~45% in LR (range: 38.7–52.7%) and ~50% in MR (range: 44.5–60.1%) (trial group B) (Table 1).

By grouping trials by risk arm distribution, comparisons of treatment intensity can be made between groups of similar proportions of the total patient population and therefore patients of similar risk. Consequently, comparisons of cumulative doses and dose intensity are made within each of the trial groups A LR, trial group A MR, trial group B LR, and trial group B MR, respectively.

### Trial group A LR

**Fig. 2:**
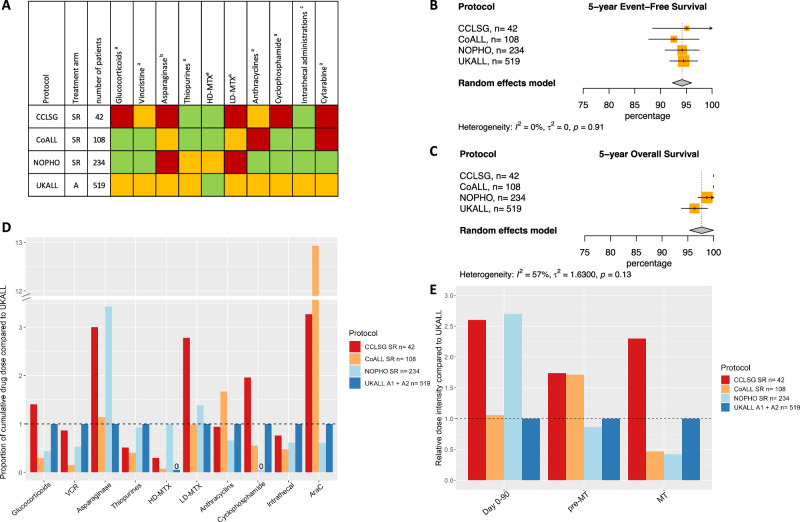
Outcome of trial group A LR. **A** Visual summary of cumulative dose analysis. Colored squares represent cumulative dose relative to the largest trial. Green represents lower cumulative dose, yellow similar, and red higher cumulative dose. **B** Meta-analysis of 5-year event-free survival (%), **C** Meta-analysis of 5-year overall survival (%) CoALL 07-03 and CCLSG ALL2004 did not have any deaths, **D** Overview of cumulative drug doses proportionate to the largest clinical trial, **E** Relative dose intensity during day 0–90, day 0 till start maintenance and maintenance therapy. VCR vincristine, HD-MTX high-dose methotrexate, LD-MTX low-dose methotrexate, AraC cytarabine, SR standard risk, MT maintenance therapy.

There was no evidence of heterogeneity between studies for survival outcomes at 5 and 10 years for trial group A LR (Figs. 2B, 2C, S4). All cumulative doses were compared to doses used in the largest trial, the MRD-guided UKALL2003 (Fig. 2A, Fig. 2D, Table 2). However, UKALL2003 did not use high-dose methotrexate (HD-MTX) in LR and therefore, cumulative HD-MTX dose was compared to the second largest trial in this trial group, i.e. NOPHO ALL2008.

Starting with comparing the MRD-guided trials, NOPHO ALL2008 used more HD-MTX and more asparaginase than UKALL2003, while using similar amounts of thiopurines. In contrast, NOPHO ALL2008 had lower cumulative doses than UKALL2003 for glucocorticoids, vincristine, anthracyclines, intrathecal administrations and cytarabine, and did not use any cyclophosphamide. Of the non-MRD-guided trials, CCLSG ALL2004 used less thiopurines and intrathecal administrations than UKALL2003 and less HD-MTX than NOPHO ALL2008, but used higher cumulative doses of glucocorticoids, low-dose methotrexate (LD-MTX), cyclophosphamide, and cytarabine. CoALL 07-03 used higher doses of cytarabine than UKALL2003 and more anthracyclines, similar amounts of asparaginase and LD-MTX, and less of all other drugs.

In trial group A LR, (with ~70% of patients in both MRD- and non-MRD guided trials), the lack of observed heterogeneity between studies means that there is little variation between studies in effect size while using different cumulative drug doses. It may be worth reevaluating the use of HD-MTX and pulses of glucocorticoids and vincristine. Moreover, using the lowest reported dose of alkylating agents (anthracyclines 80 mg/m^2^, cyclophosphamide 0 mg/m^2^) (Table 2) or number of intrathecal administrations (*n* = 10) reported might be sufficient within this group (Table 2).

### Trial group A MR

**Fig. 3:**
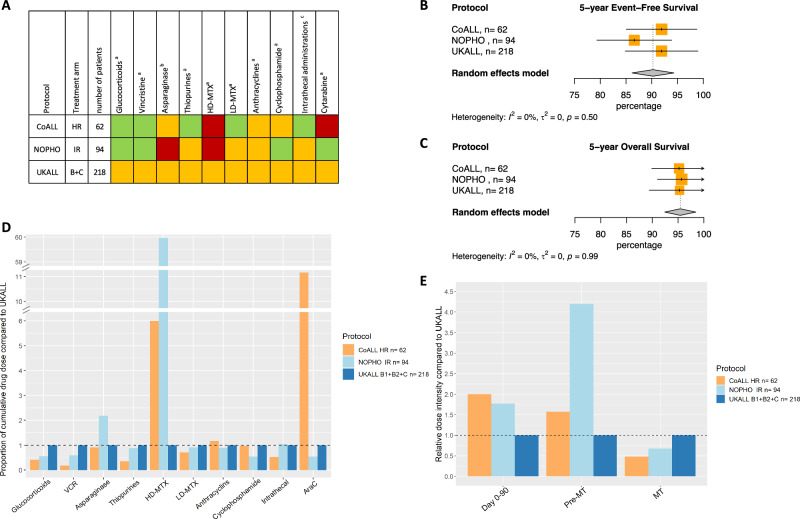
Outcome of trial group A MR. **A** Visual summary of cumulative dose analysis. Colored squares represent cumulative dose relative to the largest trial. Green represents lower cumulative dose, yellow similar, and red higher cumulative dose. **B** Meta-analysis of 5-year event-free survival (%), **C** Meta-analysis of 5-year overall survival (%), **D** Overview of cumulative drug doses proportionate to the largest clinical trial, **E** Relative dose intensity during day 0–90, day 0 till start maintenance and maintenance therapy. VCR vincristine, HD-MTX high-dose methotrexate, LD-MTX low-dose methotrexate, AraC cytarabine, SR standard risk, MT maintenance therapy.

CCLSG ALL2004 had few patients in the MR arm (*n* = 10, Table 1) therefore it was excluded from the analysis. There was no observed heterogeneity in the trials, CoALL 07-03, NOPHO ALL2008 and UKALL2003 on 5- and 10-year survival outcomes (Figs. 3B, 3C, S5). Cumulative doses were again compared to those used in the largest trial, the MRD-guided UKALL2003 (Figs. 3A, 3D, Table 2). When comparing the MRD-guided trials, NOPHO ALL2008 used more HD-MTX and asparaginase than UKALL2003, while using less glucocorticoids, vincristine, cyclophosphamide and cytarabine as observed in LR. The remaining non-MRD guided trial, CoALL 07-03, used a higher cumulative dose of HD-MTX and cytarabine, the same dose of asparaginase and alkylating agents and less of the remaining drugs compared to UKALL2003.

Jointly, as in LR, these findings suggest that in trial group A MR, encompassing ~30% of all patients in MRD- and non-MRD guided trials, pulses of glucocorticoids and vincristine in the and HD-MTX might be superfluous and there might be a possibility to use less alkylating agents.

### Trial group B LR

**Fig. 4:**
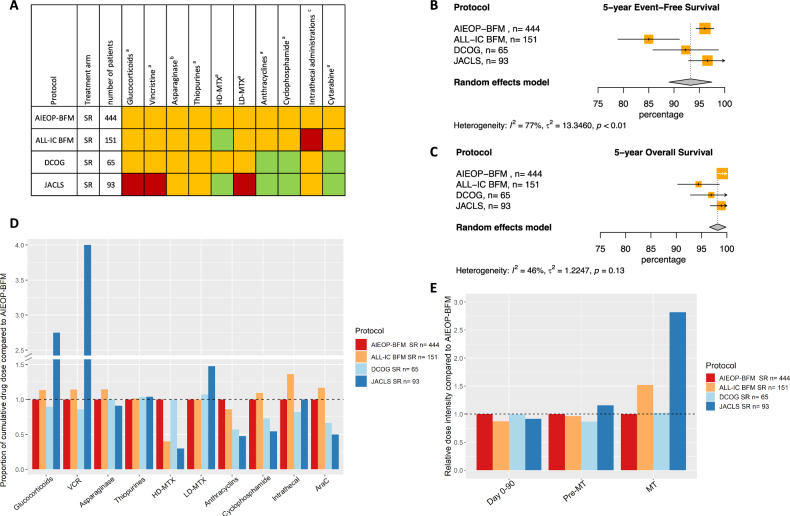
Outcome of trial group B LR. **A** Visual summary of cumulative dose analysis. Colored squares represent cumulative dose relative to the largest trial. Green represents lower cumulative dose, yellow similar, and red higher cumulative dose. **B** Meta-analysis of 5-year event-free survival (%), **C** Meta-analysis of 5-year overall survival (%), **D** Overview of cumulative drug doses proportionate to the largest clinical trial, **E** Relative dose intensity during day 0-90, day 0 till start maintenance and maintenance therapy. VCR vincristine, HD-MTX high-dose methotrexate, LD-MTX low-dose methotrexate, AraC cytarabine, SR standard risk, MT maintenance therapy.

In trial group B LR heterogeneity was present for 5-year EFS and 5- and 10-year CIR (Figs. 4B, 4C, S6, S7). This means that the outcomes examined vary between the studies. A sensitivity analysis removing ALL-IC BFM2002, the trial with the lowest 5- and 10-year EFS and 10-year CIR, was performed and did not show heterogeneity between the remaining studies (Fig. S8). When comparing the MRD-guided trials in this trial group, DCOG ALL10 used less anthracyclines, cyclophosphamide and cytarabine but similar cumulative doses of all other drugs as the AIEOP-BFM ALL2000 (Fig. 4A, 4D, Table 2). The non-MRD guided trial, JACLS ALL-02 used less of the same three drugs but also less HD-MTX while using more glucocorticoids, vincristine and LD-MTX than AIEOP-BFM ALL 2000. ALL-IC BFM ALL 2002 used less HD-MTX, but more intrathecal administrations than AIEOP-BFM ALL 2000.

Considering the arms in trial group B LR with no observed heterogeneity, encompassing ~45% of all patients in both MRD- and non-MRD guided trials, it is advisable to reassess the use of pulses with glucocorticoids and vincristine and higher doses of alkylating agents during induction or consolidation. Likewise, the possibility of reducing the cumulative dose of HD-MTX could be considered.

### Trial group B MR

**Fig. 5:**
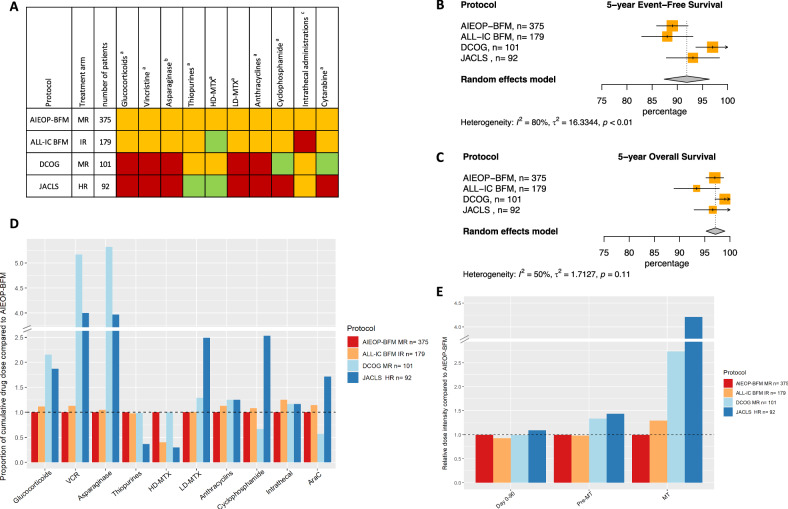
Outcome of trial group B MR. **A** Visual summary of cumulative dose analysis. Colored squares represent cumulative dose relative to the largest trial. Green represents lower cumulative dose, yellow similar, and red higher cumulative dose. **B** Meta-analysis of 5-year event-free survival (%), **C** Meta-analysis of 5-year overall survival (%), **D** Overview of cumulative drug doses proportionate to the largest clinical trial, **E** Relative dose intensity during day 0–90, day 0 till start maintenance and maintenance therapy. VCR vincristine, HD-MTX high-dose methotrexate, LD-MTX low-dose methotrexate, AraC cytarabine, SR standard risk, MT maintenance therapy.

In trial group B MR, differences in outcome were observed. Heterogeneity was observed for 5-year EFS and CIR and 10-year OS (Figs. 5B, 5C, S9, S10). A sensitivity analysis where the DCOG ALL10 trial was removed from the meta-analysis (Fig. S11) showed no statistical heterogeneity. Since there are no reasons to believe the observed heterogeneity is due to anything else than difference in outcome, we still assessed the doses used in the DCOG ALL10 trial. When comparing the MRD-guided AIEOP-BFM ALL 2000 and DCOG ALL10 trials, the latter resulted in a higher EFS and used less cyclophosphamide and cytarabine, but more glucocorticoids, vincristine, asparaginase, LD-MTX and anthracyclines. Of the non-MRD guided trials, JACLS ALL-02 had higher cumulative doses of the same five drugs and additionally more cyclophosphamide and cytarabine while using less thiopurines and HD-MTX. ALL-IC BFM ALL 2002 used less HD-MTX and had a higher number of intrathecal administrations compared to AIEOP-BFM ALL 2000 (Fig. 5A, 5D, Table 2).

Differences in outcome, cumulative doses and dose intensities show that the trials with additional asparaginase in consolidation and pulses of glucocorticoids and vincristine during maintenance therapy have the highest 5-year EFS.

### Dose intensity

All drugs analyzed in this study are used before the start of maintenance while maintenance mainly consists of LD-MTX, thiopurines and pulses of glucocorticoids and vincristine. Consequently, the differences in cumulative dose of HD-MTX, asparaginase and cytarabine between trials in group A reflect in differences in dose intensity before the start of maintenance leading to higher dose intensities for CCLSG ALL2004 LR, NOPHO ALL2008 LR and MR and CoALL 07-03 MR (Figs. 2E, 3E, S12, S13). The UKALL2003 LR and MR arms showed a higher dose intensity during maintenance due to the use of pulses of glucocorticoids and vincristine (Figs. 2E, 3E, S12, S13) while CCLSG ALL2004 LR had the highest dose intensity due to the use of pulses together with additional drugs during maintenance (Fig. 3E, S12).

In group B the higher cumulative dose of asparaginase in DCOG ALL10 MR was reflected in a higher dose intensity before start of maintenance while the use of pulses with glucocorticoids and vincristine led to a higher dose intensity during maintenance (Fig. 5E, S15). A similar pattern, however more pronounced, was seen for JACLS ALL-02 SR and MR due to the higher cumulative doses of several drugs before maintenance and the use of pulses together with additional drugs during maintenance (Figs. 4E, 5E, S14, S15).

### Site of relapse

All trials show a low percentage of relapse in LR and MR arms (Fig. 1C) and therefore the absolute number of bone-marrow and central nervous system relapse is low (Fig. S16A). The bone-marrow relapse rates are similar comparing different lengths of maintenance or number or kind of intrathecal administrations. The rate of isolated and combined central nervous system relapse, however, is lowest in trials using triple drug intrathecal administrations (Fig. S16B).

## Discussion

All trials show that *ETV6*::*RUNX1* ALL has excellent survival rates in spite of different treatment intensities, also irrespective of risk group distribution and use of MRD in stratification. These results suggest extensive over-treatment is likely and thus, treatment reduction for most patients should be considered. Since prospective testing in randomized trials is challenged by difficulties in obtaining sufficient study power, the over-treatment conundrum needs to be approached with innovative ways. This explorative research shows that trials with high cumulative drug doses or dose intensity are not necessarily those associated with better survival outcomes. For trial group A LR and MR and trial group B LR, there is a suggestion that pulses of glucocorticoids and vincristine during maintenance, HD-MTX and alkylating agents might not be necessary. Careful consideration should be given to the potential redundancy of these treatments in these specific trial groups. Further research into the association between received dose intensity and survival outcomes with novel statistical methodology is needed [32, 33]. In trial group B MR, additional asparaginase during intensification and pulses of glucocorticoids and vincristine might have contributed to better 5-year EFS and 5-year CIR. Since all trials use different combinations of cumulative dose and dose intensity of the different drugs, suggesting lower doses across several protocols might seem counterintuitive. The high survival rates observed at 5 and 10 years across all trials however, emphasize the potential for therapy reduction. This is further underlined by the results of the Children’s Oncology Group AALL0331 Study in low risk ALL of which 577 (62.1%) of the 929 included patients was *ETV6*::*RUNX1* positive showing 6-year event free survival of 94.0% (± 0.8%) and overall survival of 99.2% (± 0.3%) despite a maximum of 4 weeks of asparagine depletion, a three-drug induction and no intensive consolidation [34]. Although salvage therapy is not assessed in detail in our study, 5-year OS over 95% in all trials (except for ALL-IC 2002) points towards effective salvage in these study groups, again affirming our argument for the possibility of treatment reduction.

Previous studies have shown pulses of glucocorticoids and vincristine during maintenance therapy may have added value in protocols with a less intensive backbone for childhood ALL in general, although to what extent is unclear [35–38]. A Chinese randomized trial in LR patients of which 67% was *ETV6*::*RUNX1* positive showed no survival advantage of pulses during maintenance therapy after an intensive backbone [39]. Since both DCOG ALL10 and AIEOP-BFM ALL 2000 have intensive induction and post-remission therapy, the difference in EFS in the MR arms might, besides differences in use of asparaginase and pulses, be due to a larger part of DCOG ALL10 patients being stratified in MR, 60.1% vs. 44.5% respectively, thereby including more “lower risk” patients in the MR arm. However, a better outcome for the DCOG ALL10 LR including 38.7% of patients vs. 52.7% in AIEOP-BFM ALL 2000, would also be expected in that case, since only the lowest risk patients are in LR, but this was not found. Since OS is similar for both MR arms, our data suggests prospective testing of two possible treatment strategies: one with more asparaginase and pulses during maintenance leading to less relapse, albeit overtreating some patients, and one without this giving many patients the opportunity for less intensive treatment, while needing to salvage more relapses.

The JACLS ALL-02 and CCLSG ALL2004 trials both used several additional drugs during maintenance therapy besides pulses of glucocorticoids and vincristine, thiopurines and LD-MTX. As shown in other studies [40, 41], trials using this late intensification did not have better survival, suggesting that these additional drugs might be omitted during maintenance therapy. Duration of exposure to and especially truncation of asparaginase [22, 42–44] is known to be associated with survival in ALL in general but also in *ETV6*::*RUNX1* ALL specifically, however the optimal duration is unclear and may differ between genetic subtypes with *ETV6*::*RUNX1* ALL being one of the most sensitive [45–47]. The NOPHO ALL2008 study showed that less than 10 weeks exposure to asparaginase leads to higher 7-year cumulative incidence of relapse compared to over 16 weeks of exposure [44] supporting our observations on asparaginase in the *ETV6*::*RUNX1* trial group B MR arms. However, in trial group A, NOPHO ALL2008 is the only trial using over 16 weeks of asparaginase, while all trials have similar survival. Similar outcome was also observed in patients treated with four vs. eight weeks of asparagine depletion in the Children’s Oncology Group AALL0331 trial in low risk ALL of which 62.7% was *ETV6*::*RUNX1* positive [34].

The effect of HD-MTX on the occurrence of CNS relapse has been debated for many years [48–50]. *ETV6*::*RUNX1* ALL specifically, has been shown to accumulate less intracellular MTX polyglutamates and therefore might benefit from the higher extracellular MTX concentrations HD-MTX provides [51]. However, our data shows that protocols using lower doses of MTX lead to similar outcome as those using HD-MTX. Of interest, our results show, in line with previous studies [52, 53], that triple IT therapy might lead to fewer CNS relapse compared to MTX only IT, while not showing improved survival.

Anthracyclines are among the most toxic drugs used during ALL treatment [11]. This study shows potential for reduction for most *ETV6*::*RUNX1* ALL patients. This finding is supported by DCOG ALL10 and ALL11 trials and randomizations within the AIEOP-BFM ALL 2000 using a cumulative dose as low as 120 mg/m2 in LR patients with similar survival rates [6, 54, 55]. In the DCOG ALL9 trial no anthracyclines were used in the LR treatment arm yielding a 5-year EFS of 95% (95% CI: 91.1–98.9%) in *ETV6*::*RUNX1* ALL [56]. This trial, however, did use many pulses of vincristine and corticosteroids leading to a cumulative vincristine dose of 68 mg/m^2^. The more recent Ma-Spore ALL 2010 trial did not use anthracyclines in LR while administering a cumulative vincristine dose of 28.5 mg/m^2^ leading to a 10-year EFS of 95% with 20% of this cohort being *ETV6*::*RUNX1* ALL [57]. The ALLTogether consortium is currently testing elimination of anthracyclines for LR patients in a randomized trial (www.clinicaltrials.gov: NCT04307576).

The lower survival outcome observed in the ALL-IC BFM ALL 2002 trial may reflect the effect of not using MRD, a powerful prognostic tool [58, 59], in risk stratification. This effect is apparent in the more recent ALL-IC BFM ALL 2009 trial which used similar therapy intensity to ALL-IC BFM ALL 2002 but implemented MRD-guided risk stratification which resulted in better survival [60]. In addition, other non-identified factors may contribute to the slightly lower outcome in the ALL-IC BFM trials mainly conducted in Eastern European and South American countries. Even within an identical protocol such as the Interfant-06 trial, the outcome in these countries is slightly lower than in West-European and North American countries [61].

There are several limitations to our study. Different methods for quantifying MRD and different cut-offs in MRD-guided trials may also have had an impact in group composition and comparability. Individual patient data on administered drugs and MRD levels would be ideal to address the relation between dose and survival outcomes. However, individual patient data was not available. To gain a better understanding about the relation between drug doses, MRD and survival, future studies using data from large study centers with available individual patient data should be performed with novel methodology as has been done for osteosarcoma [32, 33]. Additionally, we analysed drug exposure per protocol and not prescribed or administered dose. Although deviations from protocol can occur, we have no reason to believe this pattern differs between the included trials. Other trial aspects such as size did show a large difference. Moreover, despite different MRD cutoffs used for stratification and thus differences in group size, EFS for LR is very similar in all four MRD guided trials (AIEOP-BFM ALL 2000, DCOG ALL10, NOPHO ALL2008 and UKALL2003 [Table 1]). This supports previous research showing the ideal MRD cutoff for *ETV6*::*RUNX1* ALL stratification is relatively high (0.01%) [62].

With current excellent survival rates for *ETV6*::*RUNX1* ALL, therapy reduction becomes an increasingly important topic. Survival outcomes observed at 5 and 10 years of eight trials show that high survival rates are possible despite quite different doses for a number of chemotherapeutic agents in contemporary trials. Our study illustrates, by looking into many protocols, that over-treatment of *ETV6*::*RUNX1* ALL must be present. Although current trials do test de-escalation of several drugs, courage is needed to implement it in standard of care. This large subset of ALL patients is worth taking a risk however, as relapse treatment is effective in the vast majority of the few that need it, while reduced treatment intensity would benefit all. When considering the proposed treatment reductions for prospective trials, more knowledge is needed about biological factors associated with relapse in a small number of *ETV6*::*RUNX1* ALL patients such as genetics beyond the translocation itself. Knowledge of these biological factors may yield opportunities to adjust future therapy more precisely early in the treatment and might give the majority of *ETV6*::*RUNX1* patients with a low risk of relapse the possibility of less intensive treatment, while not undertreating the few patients with a higher risk of relapse.

### Supplementary information

Appendix

## Data Availability

Data is available upon request from the corresponding author.
