# Supplementary material for: ETV6::RUNX1 Acute Lymphoblastic Leukemia: how much therapy is needed for cure?
Source: Leukemia. 2024 Jun 6;38(7):1477–87. doi: 10.1038/s41375-024-02287-7 (PMC11216990; doi:10.1038/s41375-024-02287-7)
Supplement: Supplementary file 1 — Appendix [file 41375_2024_2287_MOESM1_ESM.docx]

**Supplementary information**

***ETV6*::*RUNX1* ALL: How much therapy is needed for cure?**

Østergaard, A *et al*.**Supplementary figures**

Figure S1. Calculation of relative dose intensity.

Abbreviations: sRDI, single drug relative dose intensity; cRDI, combined relative dose intensity.


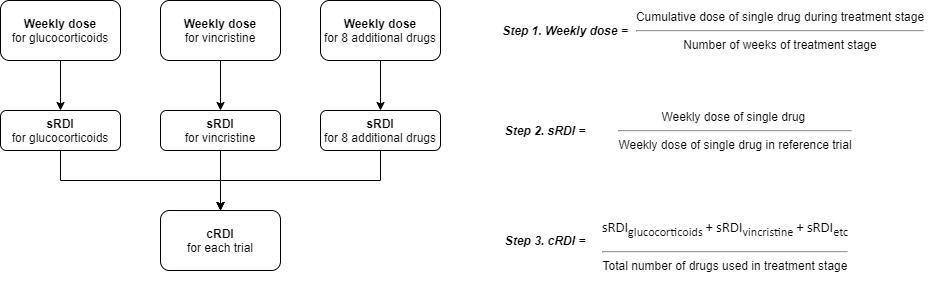


Figure S2. Meta-analysis of 5-year survival outcomes for LR and MR arms. **A** Event-Free Survival, **B** Overall Survival, **C** Cumulative Incidence of Relapse **D** Death in Complete Remission

Abbreviations: EFS, Event-Free Survival; CI, confidence interval; OS, Overall Survival; CIR, Cumulative Incidence of Relapse; DCR, Death in Clinical Remission.

Figure S3. Meta-analysis of 10-year survival outcomes for LR and MR arms. **A** Event-Free Survival, **B** Overall Survival, **C** Cumulative Incidence of Relapse **D** Death in Complete Remission

Figure S4. Meta-analysis survival outcomes for trial group A LR arms. **A** 5-year Event-Free Survival, **B** 5-year Cumulative Incidence of Relapse, **C** 10-year Event-Free Survival **D** 10-year Cumulative Incidence of Relapse

Figure S5. Meta-analysis of 5-year survival outcomes for group A MR arms. **A** Event-Free Survival, **B** Overall Survival, **C** Cumulative Incidence of Relapse **D** Death in Complete Remission

Figure S6. Meta-analysis of 5-year survival outcomes for trial group B LR arms. **A** Event-Free Survival, **B** Overall Survival, **C** Cumulative Incidence of Relapse **D** Death in Complete Remission

Figure S7. Meta-analysis of 10-year survival outcomes for trial group B LR arms. **A** Event-Free Survival, **B** Overall Survival, **C** Cumulative Incidence of Relapse **D** Death in Complete Remission

Figure S8. Sensitivity analysis for B LR arms. **A** 5-year Event-Free Survival, **B** 5-year Cumulative Incidence of Relapse, **C** 10-year Cumulative Incidence of Relapse

Relapse

Figure S9. Meta-analysis of 5-year survival outcomes of trial group B MR arms. **A** Event-Free Survival, **B** Overall Survival, **C** Cumulative Incidence of Relapse **D** Death in Complete Remission

Figure S10. Meta-analysis of 10-year survival outcomes for trial group B MR arms. **A** Event-Free Survival, **B** Overall Survival, **C** Cumulative Incidence of Relapse **D** Death in Complete Remission

Figure S11 Sensitivity analysis of survival outcomes of trial group B MR arms. **A** 5-year Event-Free Survival, **B** 5-year Cumulative incidence of Relapse, **C** 10-year Overall Survival

Figure S12 Outcome of relative dose intensity calculations for group A Low Risk arms. **A** First 90-days dose intensity relative to UKALL, **B** Pre-maintenance therapy dose intensity relative to UKALL, **C** Maintenance therapy dose intensity relative to UKALL, **D** Absolute values of calculated dose intensity relative to UKALL.

Abbreviations: VCR, vincristine; HD-MTX, high-dose methotrexate; LD-MTX, low-dose methotrexate; AraC, cytarabine; SR, standard risk; sRDI, single drug relative dose intensity; cRDI, combined drug relative dose intensity.


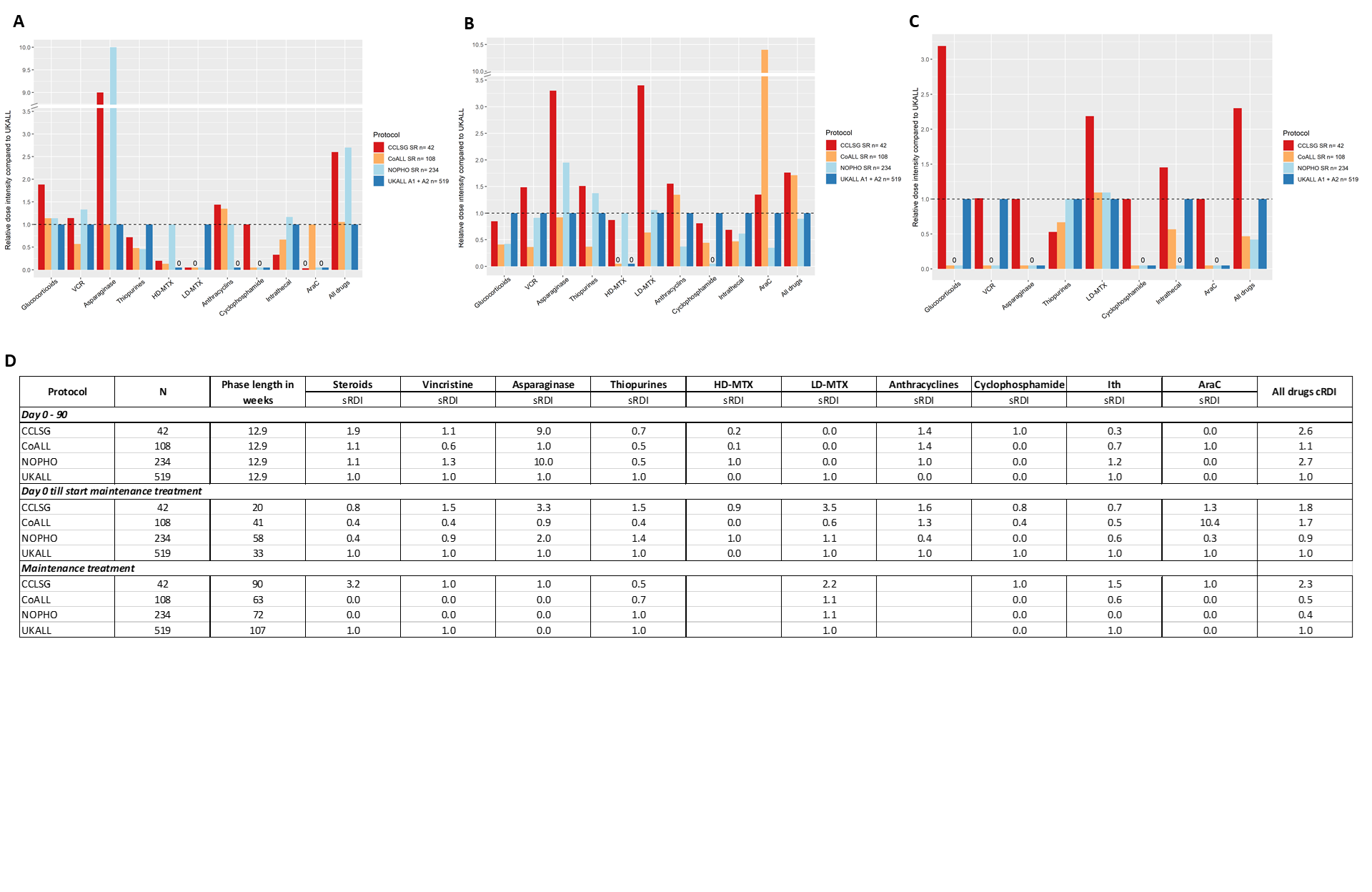


Figure S13. Outcome of relative dose intensity calculations for group A Medium Risk arms. **A** First 90-days dose intensity relative to UKALL, **B** Pre-maintenance therapy dose intensity relative to UKALL, **C** Maintenance therapy dose intensity relative to UKALL, **D** Absolute values of calculated dose intensity relative to UKALL.

Abbreviations: VCR, vincristine; HD-MTX, high-dose methotrexate; LD-MTX, low-dose methotrexate; AraC, cytarabine; IR, intermediate risk; HR, high risk; sRDI, single drug relative dose intensity; cRDI, combined drug relative dose intensity.


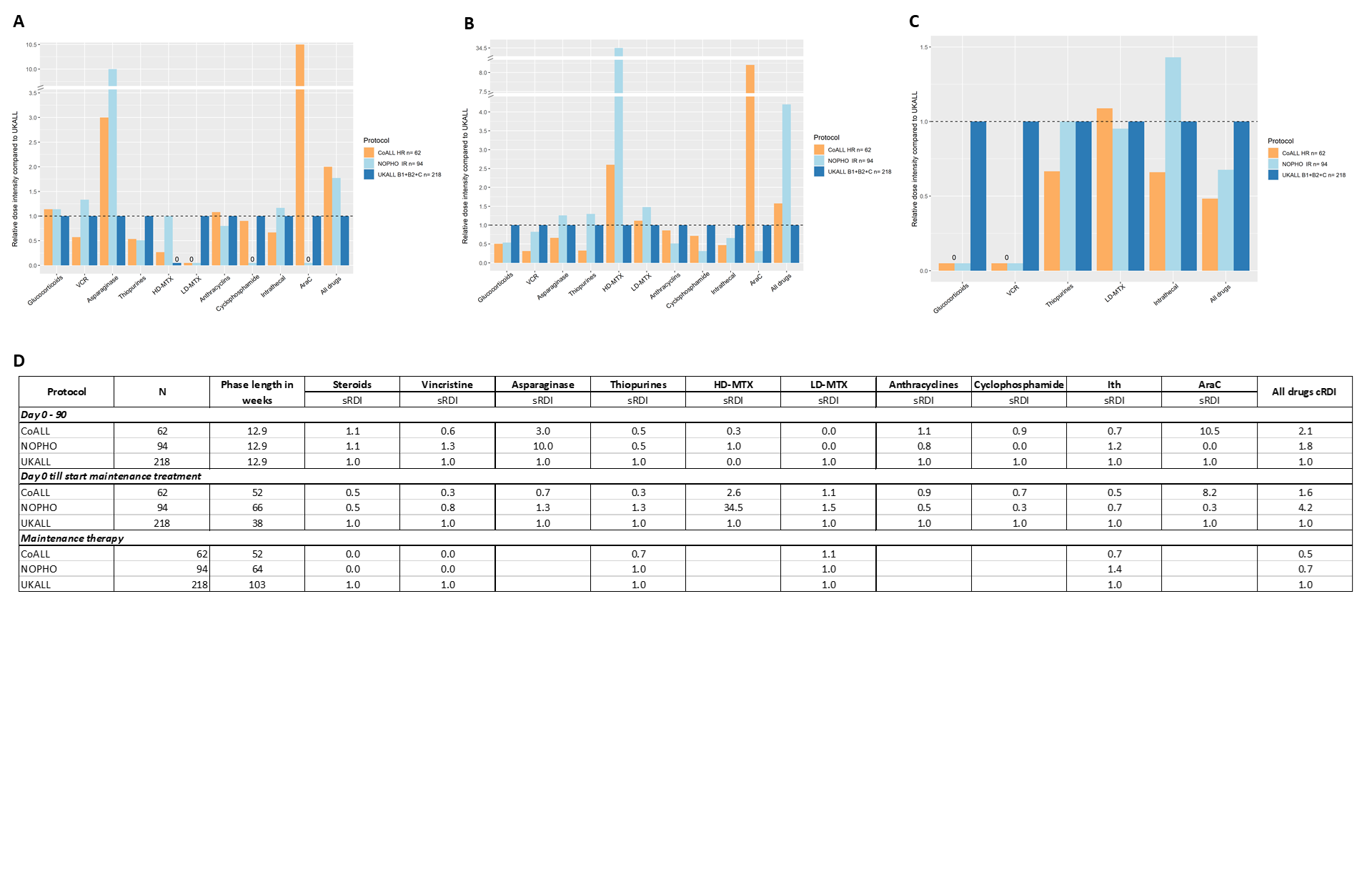


Figure S14. Outcome of relative dose intensity calculations for group B Low Risk arms. **A** First 90-days dose intensity relative to AIEOP-BFM, **B** Pre-maintenance therapy dose intensity relative to AIEOP-BFM, **C** Maintenance therapy dose intensity relative to AIEOB-BFM, **D** Absolute values of calculated dose intensity relative to AIEOP-BFM.


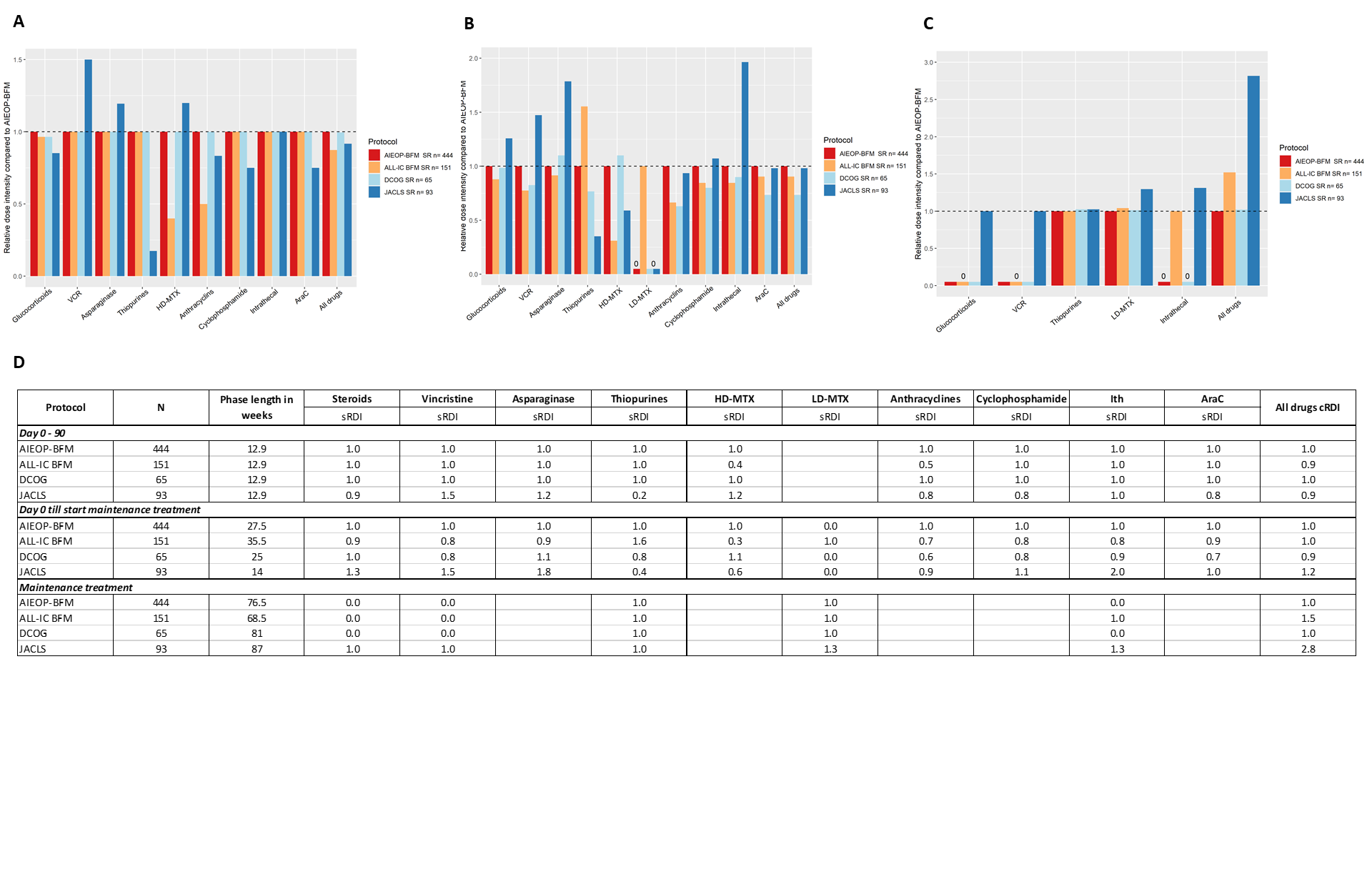
Abbreviations: VCR, vincristine; HD-MTX, high-dose methotrexate; LD-MTX, low-dose methotrexate; AraC, cytarabine; SR, standard risk; sRDI, single drug relative dose intensity; cRDI, combined drug relative dose intensity.

Figure S15. Outcome of relative dose intensity calculations for group B Medium Risk arms. **A** First 90-days dose intensity relative to AIEOP-BFM, **B** Pre-maintenance therapy dose intensity relative to AIEOP-BFM, **C** Maintenance therapy dose intensity relative to AIEOP-BFM, **D** Absolute values of calculated dose intensity relative to AIEOP-BFM.


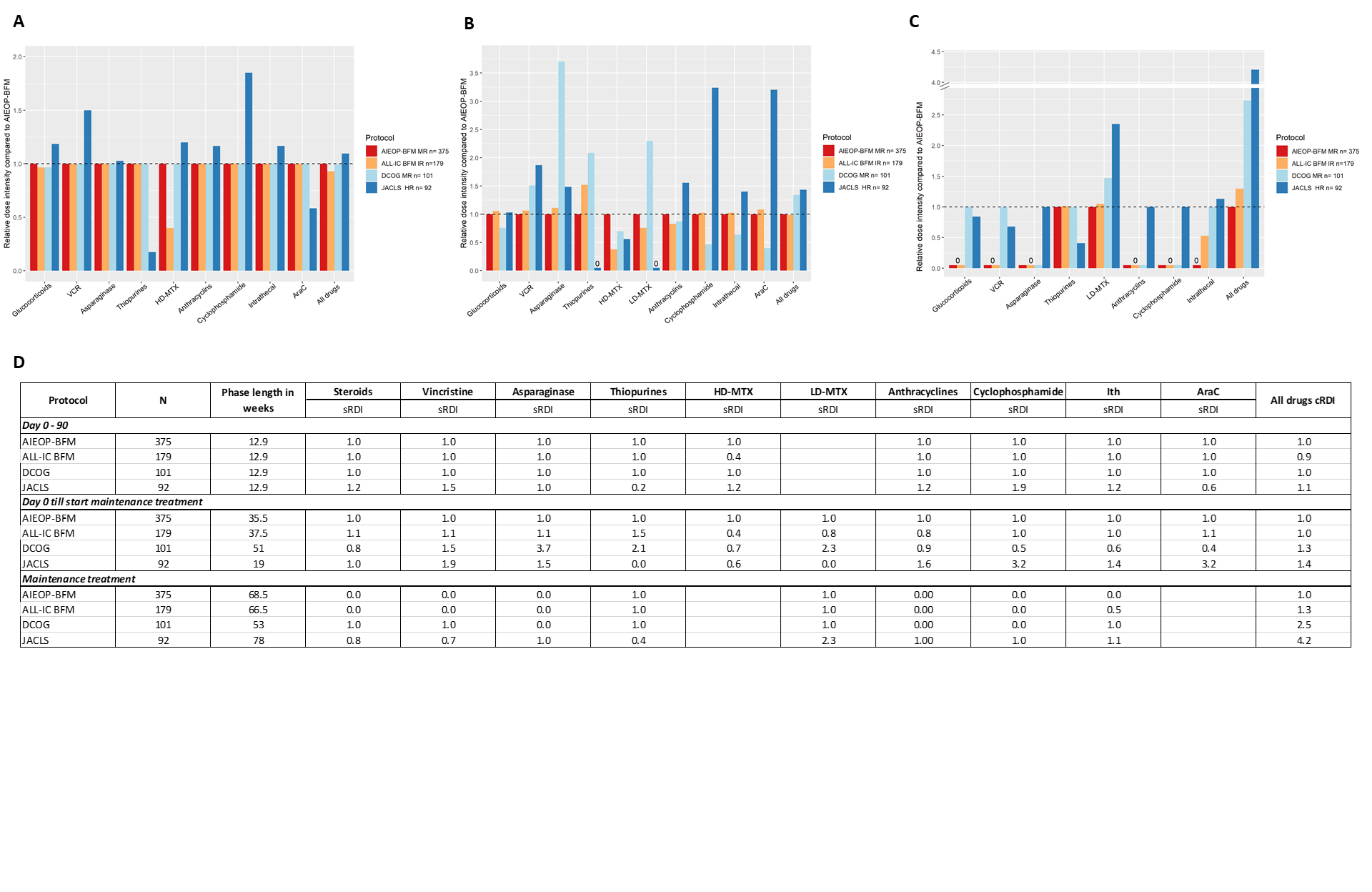
Abbreviations: VCR, vincristine; HD-MTX, high-dose methotrexate; LD-MTX, low-dose methotrexate; AraC, cytarabine; MR, medium risk; HR, high risk; sRDI, single drug relative dose intensity; cRDI, combined drug relative dose intensity.

Figure S16 **A** Relapse in clinical trials included in *ETV6*::*RUNX1* therapy intensity study. **B** Number of intrathecal administrations plotted against the percentage of patients with relapse with central nervous system involvement.

*Mean per protocol length of maintenance therapy for low and medium risk patients

Abbreviations: CNS, central nervous system; MTX, methotrexate.

**Supplementary tables**

Table S1. Risk group distribution and 10-year outcome of trials included in *ETV6*::*RUNX1* therapy intensity study.

Abbreviations: LR, low risk; MR, medium risk; HR, high risk; EFS, event-free survival; OS, overall survival; CIR, cumulative incidence of relapse; DCR, death in complete remission; CCLSG, Children’s Cancer and Leukemia Study Group; CoALL, Childhood Acute Lymphoblastic Leukemia; NOPHO, Nordic Society of Paediatric Haematology and Oncology; UKALL, United Kingdom Acute Lymphoblastic Leukaemia; AIEOP-BFM, Associazione Italiana di Ematologia e Oncologia Pediatrica and Berlin Frankfurt Münster; ALL IC-BFM, Acute Lymphoblastic Leukemia Intercontinental-Berlin Frankfurt Münster; DCOG, Dutch Childhood Oncology Group; JACLS, Japan Childhood Leukemia Study Group; LR, low risk; MR, medium risk; HR, high risk.
